# Reduced focal fiber collinearity in the cingulum bundle in adults with obsessive-compulsive disorder

**DOI:** 10.1038/s41386-019-0353-4

**Published:** 2019-02-25

**Authors:** A. Versace, S. Graur, T. Greenberg, J. P. Lima Santos, H. W. Chase, L. Bonar, R. S. Stiffler, R. Hudak, Tae Kim, A. Yendiki, B. Greenberg, S. Rasmussen, H. Liu, S. Haber, M. L. Phillips

**Affiliations:** 1 Department of Psychiatry, Western Psychiatric Institute and Clinic, University of Pittsburgh Medical Center, University of Pittsburgh, Pittsburgh, PA USA; 2 Department of Radiology, University of Pittsburgh Medical Center, University of Pittsburgh, Pittsburgh, PA USA; 3 Athinoula A. Martinos Center for Biomedical Imaging, Department of Radiology, Massachusetts General Hospital, Harvard Medical School, Boston, MA USA; 4 Department of Psychiatry and Human Behavior, Brown Medical School, Butler Hospital and Providence VA Medical Center, Providence, RI USA; 5 Department of Pharmacology and Physiology, University of Rochester Medical Center, Rochester, NY 14642 USA

**Keywords:** Depression, Reward

## Abstract

Obsessive-compulsive disorder (OCD) is a disabling condition, often associated with a chronic course. Given its role in attentional control, decision-making, and emotional regulation, the anterior cingulate cortex is considered to have a key role in the pathophysiology of the disorder. Notably, the cingulum bundle, being the major white matter tract connecting to this region, has been historically a target for the surgical treatment of intractable OCD. In this study, we aimed to identify the extent to which focal—more than diffuse—abnormalities in fiber collinearity of the cingulum bundle could distinguish 48 adults with OCD (mean age [SD] = 23.3 [4.5] years; F/M = 30/18) from 45 age- and sex-matched healthy control adults (CONT; mean age [SD] = 23.2 [3.8] years; F/M = 28/17) and further examine if these abnormalities correlated with symptom severity. Use of tract-profiles rather than a conventional diffusion imaging approach allowed us to characterize white matter microstructural properties along (100 segments), as opposed to averaging these measures across, the entire tract. To account for these 100 different segments of the cingulum bundle, a repeated measures analysis of variance revealed a main effect of group (OCD < CONT; F_[1,87]_ = 5.3; *P* = 0.024) upon fractional anisotropy (FA, a measure of fiber collinearity and/or white matter integrity), in the cingulum bundle, bilaterally. Further analyses revealed that these abnormalities were focal (middle portion) within the left and right cingulum bundle, although did not correlate with symptom severity in OCD. Findings indicate that focal abnormalities in connectivity between the anterior cingulate cortex and other prefrontal cortical regions may represent neural mechanisms of OCD.

## Introduction

Obsessive-compulsive disorder (OCD), characterized by intrusive thoughts and time-consuming compulsive behaviors [1], is often associated with a chronic and severe course that can lead to severe social and functional impairments [1]. The prevalence of OCD was previously underestimated [1]; more recent estimates indicate a lifetime prevalence of 2.3% in the general population [1], and a high comorbidity with other psychiatric disorders, including anxiety, depression, impulse-control, and substance use disorders [2]. While selective serotonin reuptake inhibitors (SSRIs) and behavioral therapy are effective treatments for the disorder, ~30% of patients with OCD do not respond adequately to conventional therapies [3].

As a result of the failure of a sizeable proportion of the OCD population to respond to conventional treatments, focal lesions (for review see ref. [4]), and, more recently, high-frequency stimulation of neural regions implicated in the pathophysiology of OCD have been advocated as viable alternatives for treatment-resistant patients with OCD [5]. One neural region that has historically been a target for focal lesions as a treatment for OCD is the anterior cingulate cortex (ACC), given its role in attentional control, decision-making, and emotional regulation [6], processes that are abnormal in OCD [7].

A prospective analysis of 198 patients with bilateral lesions of the ACC—bilateral cingulotomy—showed lasting improvements in approximately half of those treated for OCD [8], where improvements persisted for at least 2 years [8]. The relief that has been reported by OCD (and other psychiatric) patients after cingulotomy is striking [9]. Interestingly, clinical improvement does not appear to originate from a direct effect of the cingulotomy procedure on reducing OCD symptom severity, but rather result from a reduction of attention to negative emotional thoughts [10]. Indeed, one study reported that negative emotional and obsessional thoughts still occurred in individuals with OCD after cingulotomy, but that such thoughts were no longer bothersome [11]. A clearer understanding of the neural mechanisms underlying OCD symptomatology is needed to elucidate the more focal neural abnormalities that reflect pathophysiologic mechanisms of OCD, and that can act as targets to guide and improve intervention strategies for the treatment of this debilitating disorder.

One way forward to increase understanding of the neural mechanisms underlying OCD is to focus more on the neural circuitry of OCD by examining the key white matter tracts that connect the ACC with other neural regions shown to be functionally abnormal in OCD. These regions include prefrontal cortical regions, such as those implicated in attentional control and set shifting (e.g., dorsolateral and ventrolateral prefrontal cortex) [12] or in value encoding (i.e., orbitofrontal cortex) [13], as well as subcortical regions primarily involved in mediating the function of the ACC and prefrontal cortical regions via fronto-striatal-thalamic-frontal circuits [14]. The combination of functional abnormalities in the ACC and these regions is thought to be associated with the difficulty in disengaging from otherwise non-salient stimuli or contexts, i.e., deficits in set shifting, and the development of obsessional thoughts and compulsive behaviors that characterize the disorder [7].

A white matter tract that carries fibers from all of these regions is the cingulum bundle, one of the most distinctive long associative tracts [6, 15, 16]. Indeed, a large number of diffusion imaging studies has consistently reported white matter structural abnormalities in the cingulum bundle in individuals with OCD (for a recent review of 17 OCD studies see ref. [17]; for a recent meta-analysis of 22 OCD studies see ref. [18]). The majority of these studies reported lower fractional anisotropy (FA), or greater mean/radial diffusivity, reflecting lower fiber collinearity in this tract in adults with OCD relative to healthy adults. Other studies, mostly in children [19–21], reported abnormal increases in FA in this tract, indicating that important developmental abnormalities may occur in the cingulum bundle in individuals with OCD. Indeed, an age effect was reported in a more anterior portion of the cingulum, where greater reductions in FA were reported in older than younger adults [22–24]. Greater mean diffusivity (i.e., lower collinearity or FA) in the left cingulum bundle was also associated with greater severity of OCD symptoms in adults with OCD [25], and greater FA in the left cingulum bundle with better performance on directed attention (Stroop Test) and executive control (Trails Making Test) tasks [19] in children with OCD, suggest that microstructural abnormalities in this tract might underlie development of some of the symptoms of OCD.

Abnormalities in other white matter tracts have also been reported in OCD. Abnormal (decreased or increased) FA has been reported in the genu [20, 26, 27], body [28, 29], and splenium [27, 30] of the corpus callosum and/or anterior projecting fibers, including the anterior corona radiata, anterior thalamic radiation, and/or anterior limb of the internal capsule [21, 25, 31, 32]. These findings are less consistent across studies than those regarding the cingulum bundle, however. This inconsistency can in part be explained by the heterogeneity of the disorder, and methodological differences existing across studies. In this regard, it is important to note that the majority of these studies acquired relatively low-resolution diffusion imaging data, mostly fewer than 30 gradients in 1.5 Tesla scanners, and used voxel-based approaches, including voxel-based morphometric analyses or tract-based spatial statistics (TBSS). While valuable for the study of white matter microstructure across the whole brain, these approaches are of limited utility for the study of the integrity of specific white matter tracts, where tractography studies are better suited. Furthermore, the structure of the cingulum bundle is complex, comprising both short (U-fibers) and long sagittal associative fibers that link medial parts of the frontal, parietal, and temporal lobes [6, 33], and projection fibers that radiate across the tract to cortical and subcortical regions [33]. As such, averaging diffusivity measures across the entire cingulum bundle, as performed in conventional tractography studies, might not capture the complex architecture of this tract.

The goal of the present study was thus to employ, for the first time, a tract-profile approach in the study of the white matter microstructure of the cingulum bundle in adults with OCD in comparison with healthy adults. Here, we used the Automated Fiber Quantification (AFQ; https://github.com/yeatmanlab/AFQ) toolbox to automatically reconstruct the cingulum bundle in each participant in native space. AFQ allows the derivation of “tract-profiles” that measure diffusivity properties at sample positions (e.g., 100 segments) from the start to the end of each reconstructed tract. Use of tract-profiles—rather than conventional approaches measuring white matter microstructure across an entire tract—allowed us to determine the extent to which white matter microstructural abnormalities previously reported in OCD were localized to focal portions (i.e., 5 or more consecutive segments) of the cingulum bundle rather than being widespread along the entire tract. This approach also allowed us to further examine the extent to which any focal abnormalities were associated with specific OCD symptomatology. We hypothesized that adults with OCD would show significantly reduced FA in focal regions of the cingulum relative to healthy adults, and specifically in portions of the tract carrying fibers from neural regions previously shown to be functionally abnormal in OCD, including the ACC, orbitofrontal cortex, dorsolateral prefrontal cortex, and subcortical nuclei. We further hypothesized that these abnormalities would be significantly associated with OCD symptom severity, where greater FA deceases would be associated with greater symptom severity. Additional major white matter tracts were also examined in exploratory analyses.

## Methods

### Participants

Ninety-three adults including 48 adults with OCD (age range = 18–35 years mean age [SD] = 23.3 [4.5] years; F/M = 30/18) and 45 age- and sex-matched healthy adults (age range = 18–33 years; mean age [SD] = 23.2 [3.8] years; F/M = 28/17) were included in the study. There were no between-group differences in socioeconomic status (SES) and predicted IQ, as measured using the National Adult Reading Test-revised [34] (Table 1). Six out of 48 adults with OCD had a comorbid diagnosis of major depressive disorder (MDD) at the time of the scan. Recruitment period was 6/3/2015–10/20/2018.

**Table 1:** Between-group differences in demographic and clinical variables

Variables	Group	*N*						Mean	SD	*t*/*χ*^2^	df	*P* (two-tailed)
Scanner (Trio/Prisma)	CONT	5	40					—	—	0.4	1	0.836
	OCD	6	42					—	—			
Age [YY]	CONT	45						23.2	3.8	0.1	91	0.901
	OCD	48						23.3	4.5			
Sex (F/M)	CONT	28	17					—	—	0.1	1	0.978
	OCD	30	18					—	—			
SES (6 levels^c^)	CONT	1	4	24	1	9	6	—	—	4.7	5	0.456
	OCD	1	9	20	0	14	4	—	—			
Predicted full IQ	CONT	45						109.9	0.9		91	**0.387**
	OCD	48						111.1	5.8			
HRSD17	CONT	45						1.2	1.2	11.6	51^a^	**<0.001**
	OCD	48						10.8	5.6			
HAMA	CONT	45						1.0	1.2	11.5	50^a^	**<0.001**
	OCD	48						11.7	6.3	11.5	50	
Y-BOCS	CONT	45						0.1	0.7	42.1	52^a^	**<0.001**
	OCD	48						20.5	3.3	42.1	52	
OC-TCDQ harm avoidance	CONT	45						3.1	4.9	14.7	80^a^	**<0.001**
	OCD	48						23.0	7.9	14.7	80	
OC-TCDQ incompleteness	CONT	45						4.4	4.9	11.4	70^a^	**<0.001**
	OCD	48						22.7	9.9	11.4	70	
POPS	CONT	36						102.5	33.6	9.3	72	**<0.001**
	OCD	38						180.6	38.4			
Difficulty with change	CONT	36						18.2	7.3	8.4	72	**<0.001**
	OCD	38						33.9	8.7			
Emotional overcontrol	CONT	36						16.1	5.9	4.9	64	**<0.001**
	OCD	38						24.8	9.1			
Rigidity	CONT	36						28.0	11.8	6.5	72	**<0.001**
	OCD	38						47.0	13.3			
Maladaptive perfectionism	CONT	36						23.6	8.8	9.6	72	**<0.001**
	OCD	38						46.8	11.8			
Reluctance to delegate	CONT	36						18.2	7.1	7.3	72	**<0.001**
	OCD	38						31.8	8.8			
Temperament and Character Scale—Harm Avoidance: anticipatory worry1	CONT	36						8.1	2.9	7.3	72	**<0.001**
	OCD	38						3.1	3.0			
Temperament and Character Scale—Harm Avoidance: Fear of uncertainty2	CONT	36						4.1	1.5	5.3	72^a^	**<0.001**
	OCD	38						1.8	2.1	0.9		
Taking any medications [yes/no]	CONT	0/43						15.0			1	**<0.001**
	OCD	18/30										

*HRSD17* Hamilton Rating Scale for Depression – 17 items, *HAMA* Hamilton Anxiety Rating Scale, *Y-BOCS* Yale-Brown Obsessive-Compulsive Scale, *OC-TCDQ* Obsessive-Compulsive Trait Core Dimensions Questionnaire, *POPS* Pathological Obsessive-Compulsive Personality Scale

^a^Equality of variance was not assumed

^b^Missing data

^c^SES levels include partial high school, high school diploma or general equivalency degree, some college (at least one year), technical school or associates degree college diploma (bachelor’s degree), and graduate or professional degree

Levels of significance are <0.05 (in bold font)

Inclusion criteria in all participants were 18–35 years of age and right-handedness. All participants were interviewed using the Structured Clinical Interview for DSM-5. Adults with OCD diagnosis without predominant hoarding symptoms and Yale-Brown Obsessive-Compulsive Scale (Y-BOCS) [35] score >16 were included in the study. Exclusion criteria in all participants were personal/family history (1st/2nd degree relatives) of schizophrenia/schizoaffective disorder, other primary psychotic disorders, bipolar disorder; present posttraumatic stress disorder; present suicidal ideation; present psychotic symptoms; personal history of head injury, neurological, neurodevelopmental (e.g., autism), tic disorders, systemic medical (metabolic, endocrine, chronic inflammatory, vascular, autoimmune) disease from medical records and self-report, all of which may confound interpretation of neuroimaging measures; MMSE score <24; premorbid IQ estimate <85; visual disturbance (<20/40 corrected Snellen visual acuity); left/mixed handedness; current, and history in the last 3 months of, alcohol and illicit substance abuse/dependence, determined by urine screen and clinical assessment of alcohol and substance use; current suicidal ideation, as assessed using the Hamilton Rating Scale for Depression, contraindications to MRI: metallic foreign objects, e.g., aneurysm clips/ pacemakers, or questionable history of metallic fragments, prone to panicking in enclosed spaces; positive pregnancy test for females/self-reporting of pregnancy. In adults with OCD, additional exclusion criteria were Y-BOCS score <16; predominant hoarding symptoms, given that hoarding may have a distinct neural circuitry [36]. In healthy adults, additional exclusion criteria were personal history of Axis I disorder, or substance abuse/dependence, family history (1st/2nd degree relatives) of OCD, neurodevelopmental disorder, schizophrenia and/or schizoaffective disorder, primary psychotic disorder, bipolar disorder, and present PTSD.

### Symptom assessment

Type and severity of depressive, anxiety and obsessive-compulsive symptoms were measured using clinical rating scales and self-report questionnaires (for more details, see Supplemental Material)

Eighteen out of 48 adults with OCD were taking medications at the time of the study. These included antidepressant medications (SSRIs). The study was approved by the Institutional Review Board of the University of Pittsburgh. After being informed on the nature and aims of the study, all participants signed the consent form before participating in the study procedures.

### Data analysis

#### Neuroimaging

Images were acquired on a 3T Siemens Magnetom Prisma (42 OCD and 40 CONT) and a 3T Siemens Magnetom Trio (6 OCD and 5 CONT) at the Magnetic Resonance Research Center, University of Pittsburgh Medical Center Health System, USA. Parameters of the dMRI acquisition are described in the Supplemental Material. Diffusion-weighted images were corrected for eddy current, subject motion and EPI distortion using *topup* and *eddy* [37, 38], within FMRIB’s Software Library (FSL). Six movement parameters, including average volume-by-volume translation and rotation in the *x*, *y*, and *z* plane, were computed as previously proposed [39] in each participant. Between-group *t*-tests did not reveal any significant difference (Supplemental Table 1).

Data analysis and reconstruction of the cingulum tractogram were performed using the freely available Automated Fiber Quantification (AFQ) package (https://github.com/yeatmanlab/AFQ/wiki). The tensor model was fitted to the *b* = 1000 data to compute FA. AFQ uses a multi-step reconstructing procedure, including whole brain tractography, tract segmentation based on well-validated tracking protocols, tract refinement based on a probabilistic fiber tract atlas (Supplemental material for more details), and tract-profile calculation. The FA values are averaged in cross-sections (i.e., segments) along the left and right cingulum bundle in each participant. For a detailed characterization of FA along the tract, we used 100 segments [40]. Other tensor-based measures, namely axial and radial diffusivity (AD, RD), represent the displacement of water along the principal and non-principal diffusion directions, and can help interpret FA abnormalities [41]. Thus, AD and RD were also extracted along the left and right cingulum bundle in each participant.

In this tract of interest approach, left and right corticospinal tracts were examined as control tracts. While the focus of this study was on the cingulum bundle, additional major white matter tracts, including forceps minor/forceps major of the corpus callosum, anterior thalamic radiation, inferior longitudinal fasciculus, parietal and temporal portion of the superior longitudinal fasciculus and uncinate fasciculus were examined in exploratory analyses.

#### Statistical approach

Demographic, clinical, and diffusion imaging measures were imported into SPSS(v24) to test main hypotheses and exploratory analyses. Given the known effect of age (linear effect) and sex on white matter, these variables were covariates in all analyses. Scanner was an additional covariate in analyses.

To test our hypotheses, we adopted the following analytic approach.

##### Level-1 analysis

Using a two-way factorial repeated measure analyses of covariance (ANCOVA), we examined the main effect of group (OCD and CONT) on FA of the left and right cingulum bundle. Specifically, LATERALITY with 2 levels (1. LEFT; 2. RIGHT) and SEGMENTS with 100 levels were the within subject factors.

##### Level-2 analysis

Post-hoc ANCOVAs were employed in adults with OCD versus CONT on FA in each segment showing a significant effect of GROUP or GROUP-related interaction in Level-1 analyses. To account for multiple comparisons of all 200 segments, i.e., the 100 × 2 segments extracted from the tract-profiles of the left and right cingulum bundles, a method for multiple hypothesis testing for possibly dependent tests was used. Specifically, to account for the possible collinearity existing between FA values of consecutive segments along a given tract, the Beta-Binomial Sequential Goodness of Fit (BBSGoF; *k*_min_ = 5, *k*_max_ = 8, blocks = 5) [42] was used, as proposed in the R package (https://cran.r-project.org/web/packages/sgof/sgof.pdf).

To better understand the nature of FA abnormalities in segment clusters showing a main effect of group upon FA in Level-2 analyses, mean AD and RD were extracted. Parallel ANCOVAs were used to examine a main effect of group upon these measures after accounting for age and gender.

#### Additional analyses

Mean FA was extracted in the segment clusters, i.e., clusters of contiguous segments within the left or right tract-profile of the cingulum bundle that showed a main effect of group or a main (simple or triple) interaction in Level-1 analysis, and that survived multiple comparison correction in Level-2 analysis. In adults with OCD, we then examined relationships between symptom severity (HRSD, HAMA, Y-BOCS, OC-TCDQ-incompleteness, OC-TCDQ-harm avoidance, and POPS in adults with OCD) and mean FA in segment clusters showing a significant main effect of group in Level-2 analyses, using six parallel repeated measure ANCOVAs, one for each symptom dimension as the between-subjects effect, and FA in the two segment clusters (LATERALITY: 2 levels, i.e., the two segment clusters in the left and right cingulum bundle) as the within-subjects effect. Significant findings were then further examined using Pearson and/or Spearman correlations, as appropriate. The effect of SSRI (ON/OFF) or the effect of MDD comorbidity (yes/no) on FA in these segment clusters were also explored, using a similar approach. To account for multiple comparisons of FA-symptom dimension severity/medication in analyses focusing just on adults with OCD (i.e., analyses using the HRSD, HAMA, Y-BOCS, OC-TCDQ-incompleteness and OC-TCDQ-harm avoidance, POPS, MDD comorbidity, and SSRIs) the significance threshold was set at *P* = 0.05/8 = 0.006.

#### Control tracts

Level-1-2 analyses were repeated for the left and right corticospinal tracts. To account for the examination of two white matter tracts in main analyses, the significance level was set at *P* = 0.05/2 = 0.025 in Level-1 analysis.

#### Other tracts

To explore the role of other major white matter tracts in the pathophysiology of OCD, Level-1-2 analyses were repeated for the left (50 segments) and right (50 segments) portion of the forceps minor and forceps major of the corpus callosum, the left and right anterior thalamic radiation, the left and right inferior longitudinal fasciculus, the left and right parietal and temporal portion of the superior longitudinal fasciculus, and the left and right uncinate fasciculus.

Scanner, age, and sex were covariates in all analyses.

#### Sensitivity analysis

Level-1-2 analyses were repeated after exclusion of data collected on the Trio.

## Results

### Demographic and clinical characteristics

There were no significant group differences in age, sex ratio, SES, and predicted IQ. As expected, adults with OCD had significantly greater anxiety (HAMA), depressive (HRSD17) and OCD (Y-BOCS, OC-TCDQ-incompleteness, OC-TCDQ-harm avoidance, and POPS) symptom severity than CONT. In addition, 16 out of 44 adults with OCD were taking SSRIs (Table 1).

### Neuroimaging

#### Level-1 analyses

After accounting for effects of scanner, age and sex, a repeated measure ANCOVA revealed a significant main effect of GROUP (between-subjects effect: F_[1,87]_ = 5.3; *P* = 0.024) on FA on the left and right cingulum bundle. This analysis also revealed a trend effect of LATERALITY (within-subjects effect: F_[1,87]_ = 3.9; *P* = 0.050; which did not survive correction for multiple comparisons, *P* = 0.050/2 = 0.025 to account for examination of the corticospinal tract, as a control tract; see above), and an effect of SEGMENT (within-subjects effect: F_[1,87]_ = 5.4; *P* < 0.001) (Table 2).

**Table 2:** Two factorial repeated measure ANCOVA in the cingulum bundle in 48 adults with OCD and 45 healthy adults

Tests of between-subjects effects	F_[1,87]_	Sig.
Group	5.3	0.024
Scanner	16.1	<0.001
Age [YY]	8.1	0.006
Sex [M/F]	1.1	0.295
Tests of within-subjects effects	F_[1,87]_	Sig.
Laterality	3.9	0.050
Laterality * group	0.1	0.767
Tests of within-subjects effects	F_[5,426]_	Sig.
Segment	5.4	<0.001
Segment * group	1.6	0.154
Tests of within-subjects effects	F_[7,596]_	Sig.
Laterality * group * segment	1.1	0.364

Age and gender were covariates in all analyses

Two factorial analysis included: Laterality (2 levels: left and right) and segment (100 levels)

#### Level-2 analyses

##### Main effect of group

**Fig. 1:**
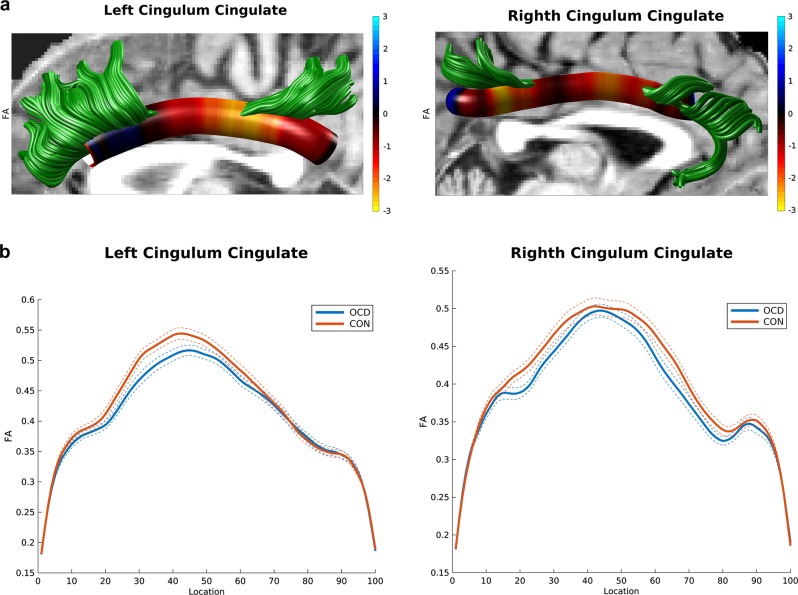
**a** 3D visualization of the left and right cingulum bundle showing lower FA in OCD versus CONT. Tractographic analyses were performed in native space. Findings are displayed in one participant to represent between-group differences (red-yellow color bar depicts significance levels of 3 > *t* > −3). **b** Curve-trajectory plots represent the mean FA along the left and right cingulum bundle in OCD versus CONT. One hundred segments were used to define the tract-profile of the left and right cingulum in each tract separately. Confidence interval of 95% is represented with dotted lines. OCD obsessive-compulsive disorder, CONT healthy control individuals

After accounting for effects of scanner, age and sex, adults with OCD showed lower FA than CONT in the middle sections of the left and right cingulum bundle (OCD versus CONT in the left cingulum bundle: *k* = 29 consecutive segments; OCD versus CONT in the right cingulum bundle: *k* = 17 consecutive segments; *P* < 0.05). In the left cingulum bundle, 22 out of these 29 consecutive segments survived correction for multiple comparisons (*P* < 0.030). In the right cingulum bundle, 15 out of these 17 consecutive segments survived correction for multiple comparisons (*P* < 0.028; Fig. 1).

Further analyses revealed that adults with OCD showed higher RD than CONT in segment clusters of the left and right cingulum bundle; however, these findings were not significant (*P* > 0.05). There was no between-group difference in AD (*P* > 0.05).

#### Additional analyses

##### In adults with OCD

After accounting for effects of scanner, age, and sex, using six parallel repeated measure ANCOVAs (one for each symptom dimension), we did not find any relationship between mean FA in the segment clusters showing between-group differences in FA in the left or right cingulum bundle and symptom severity as measured by the HDRS-D, HAMA-A, Y-BOCS OC-TCDQ-incompleteness and OC-TCDQ-harm avoidance, and POPS in adults with OCD. We also did not find any effect of MDD comorbidity or SSRI on mean FA in these segment clusters in the left and right cingulum bundle (Supplemental Table 2).

#### Control tracts

After accounting for the effect of age and sex, a repeated measure ANCOVA found no effect of GROUP (F_[1,91]_ = 2.7; *P* = 0.107), no effect of LATERALITY (F_[1,91]_ = 0.5; *P* = 0. 938), and no LATERALITY by SEGMENT by GROUP interaction (F_[99,5488]_ = 0.5; *P* = 0.695). Findings in the corticospinal tracts are reported in detail in Supplemental Table 3A.

#### Other tracts

In other major white matter tracts, there was a LATERALITY by SEGMENT by GROUP interaction (F_[98,7938]_ = 1.8; *P* = < 0.001) in the inferior longitudinal fasciculus. Further analyses revealed that adults with OCD showed lower FA that healthy adults in a middle portion (9 segments) of the left inferior longitudinal fasciculus. Mean FA in this segment cluster did not correlate with any symptom dimension, however (all *P* > 0.05). These findings and findings on other major white matter tracts are reported in detail in Supplemental Table 3B.

#### Sensitivity analysis

Findings are reported in the Supplemental Table 4.

## Discussion

The goal of the present study was to use, for the first time, a tract-profile approach to examine the extent to which diffusion imaging abnormalities previously reported in the cingulum bundle of adults with OCD are focal or extended across the entire cingulum bundle. In support of previous studies [17, 30, 43–47], findings from this study showed lower collinearity of fibers in both the left and right cingulum bundle of adults with OCD versus healthy adults. Using a tract-profile approach for the first time in OCD, our findings further demonstrate that lower FA in the cingulum bundle is focal, middle section of this tract. Further analyses suggested that in adults with OCD there might be a greater complexity of the fiber architecture and/or abnormal integrity of the fibers (greater RD) in comparison to healthy adults; however, this finding did not reach significance. It is probable that the focal abnormalities in this tract reflect abnormalities in the collinearity/integrity of specific fibers that connect the ACC with other prefrontal cortical regions. Indeed, fibers in these portions of the cingulum bundle have been shown to project not only to other portions of the ACC, including subgenual and dorsal ACC, but also to prefrontal cortex, including the orbitofrontal cortex, and subcortical regions, including the anterior ventral nucleus and lateral dorsal nucleus of the thalamus [6]. All these regions implicated in neural mechanisms underlying OCD [6, 7, 12, 14].

To our knowledge, only two previous diffusion imaging studies employed similar tractographic approaches in OCD [46, 48]. Neither of these studies reported diffusion imaging abnormalities in the cingulum bundle. In fact, one of these studies did not find any FA abnormality in any of the major white matter tracts examined [46]. The second study reported lower FA only in the forceps minor and in the right uncinate fasciculus [48]. Given that the cingulum bundle is a large white matter tract of ~800 2 mm^2^ voxels, it is possible that conventional approaches examining mean diffusivity parameters across the entirety of each tract might have precluded identification of any focal abnormality in the cingulum. Power might also be a factor, given that these studies included fewer than 30 patients with OCD.

As expected, we did not find any significant effect of group on FA in the corticospinal tracts in this study. In contrast with previous studies, however, examination of other major white matter tracts also did not reveal any other white matter microstructural abnormalities in adults with OCD (versus healthy adults). While the tract-profiles allow examination of white matter microstructure in segments along the length—the major axis—of a given tract, it is important to note that very large white matter tracts/regions, such as the corpus callosum or internal capsule, are known to have both a rostro-caudal and/or medial-lateral organization of fibers. For example, more ventral portions of the anterior limb of the internal capsule project to ventromedial/ventrolateral portions of the prefrontal cortical regions, while dorsal portions of the anterior limb of the internal capsule project to dorsomedial/dorsolateral portions of the prefrontal cortical regions [49] Thus, tract-profile approaches are not suitable for the study of the microstructure of white matter tracts in which rostro-caudal and/or medial-lateral segmentations are needed.

Certain limitations of the study should be considered. Additional analyses did not reveal any relationship between FA abnormalities and severity of OCD symptoms as measured by the Y-BOCS, or with severity of other symptoms in adults with OCD at the time of the scan. It is important to note, however, that this sample size allowed us to explore linear relationships. Larger studies would allow us to model polynomial functions, which, in turn, might help identify more complex relationships between FA in the anterior segments of the cingulum bundle and severity of OCD symptoms. As mentioned above, our approach did not identify abnormalities in other major white matter tracts in which abnormalities in FA have previously been reported in individuals with OCD. Studies employing protocols for segmenting major white matter tracts are needed to further understand the contribution of white matter microstructural abnormalities in other tracts in OCD.

In summary, using a tract-profile approach for the first time in OCD, we demonstrate focal abnormalities in the middle portions of the left and right cingulum bundles in adults with OCD. The middle portion of the cingulum bundle carries fibers specifically between the ACC and other prefrontal and subcortical regions that have been proposed to have a key role in the pathophysiology of OCD. New protocols are needed to allow segmentation of other white matter tracts, such as the corpus callosum and anterior thalamic radiation/internal capsule, to help elucidate more focal neural mechanisms of OCD, to ultimately guide the development of new interventions for this debilitating disorder.

## Funding and disclosure

The present study was supported by the National Institute of Mental Health grant P50 MH106435 (PI: Dr. Haber). This funding agency was not involved in the design or conduct of the study, the collection, management, analysis, or interpretation of the data, or the preparation, review, or approval of the manuscript. The authors declare no competing interests.

## Supplementary information

Supplemental Material
