# Supplementary material for: Reduced focal fiber collinearity in the cingulum bundle in adults with obsessive-compulsive disorder
Source: Neuropsychopharmacology. 2019 Feb 25;44(7):1182–8. doi: 10.1038/s41386-019-0353-4 (PMC6784994; doi:10.1038/s41386-019-0353-4)
Supplement: Supplementary file 1 — Supplemental Material [file 41386_2019_353_MOESM1_ESM.docx]

**Supplemental Material**

**Methods**

***Participants***

***Symptom Assessment.*** In adults with OCD and healthy adults, type and severity of depressive symptoms were measured using the 17 item-Hamilton Rating Scale for Depression(HRSD); (Hamilton, 1960) anxiety symptoms were measured using the Hamilton Anxiety Rating Scale (HAMA);(Hamilton, 1959) and obsessive-compulsive symptoms were measured using the Y-BOCS.(Goodman *et-al*, 1989) Additional measures were the Obsessive-Compulsive Trait Core Dimensions Questionnaire OC-TCDQ,(Ecker *et-al*, 2011) assessing the severity of different OCD behavioral dimensions, specifically, incompleteness and harm avoidance, temperamental features that are associated with OCD;(Ecker and Gonner, 2008; Ettelt *et-al*, 2008; Lyoo *et-al*, 2001; Pietrefesa and Coles, 2008) and the Pathological Obsessive-Compulsive Personality Scale (POPS).

***Data analysis***

***Neuroimaging***.

***Data Acquisition.*** Images were acquired on a 3T Siemens Magnetom Prisma or Trio (6 adults with OCD and 5 healthy adults) at the Magnetic Resonance Research Center, University of Pittsburgh Medical Center Health System, USA. A built-in whole-body coil and a 32-channel head coil were used for the RF transmission and the RF reception, respectively. A single-shot spin-echo echo planar imaging (SE-EPI) sequence was acquired with 197 optimized non-colinear diffusion-weighting gradient directions (32 volumes with b= 700 s/mm2, 65 volumes with b= 1000 s/mm^2^, 100 volumes with b= 2500 s/mm^2^) and 13 reference volumes with b=0 s/mm^2^ (repetition time (TR)=3000ms, echo time (TE)=120ms, flip angle=90, field-of-view (FOV)=256×256, 2 mm^3^ isotropic voxel, SMS factor=4, acquisition time=630 seconds). In accordance with a forward-reverse protocol, the acquisition was collected twice with opposite phase encoding directions 4(P>>A and A>>P). The sampling scheme was based on the strength and number of gradient directions for each shell to ensure optimal angular coverage across the entire sequence.(Caruyer *et-al*, 2013) The same neuroimaging protocol was collected on both Trio and Prisma scanners.

***Data Analysis.*** The Automated Fiber Quantification (AFQ; [https://github.com/yeatmanlab /AFQ/wiki](https://github.com/yeatmanlab%20/AFQ/wiki)) package was used to analyzed these data, as proposed in Yeatman at al.{Yeatman, 2012 #638}. AFQ uses a multi-step reconstructing procedure: (1) Whole brain tractography is initiated from each white matter voxel with fractional anisotropy (FA) >0.3. (2) Fibers that pass through two waypoint regions of interest (ROIs) become candidates for the fiber group, based on the Wakana’s protocols.(Wakana *et-al*, 2004) (3) Each candidate fiber is then scored based on its similarity to a standard fiber tract probability map. Fibers with high probability scores are retained. (4) Fibers tracts are represented as a 3-dimensional Gaussian distribution and outlier fibers that deviate substantially from the mean position of the tract are removed. (5) The fiber group is clipped to the central portion that spans between the two defining ROIs. (6) The fiber group core is calculated by resampling each fiber into 100 equidistant segments and calculating the mean location of each segment. Diffusion imaging properties (e.g. FA, AD, RD) are calculated at each segment by taking a weighted average of the FA measurements (or other diffusion properties) for each individual fiber at that segment. Weights are determined based on the Mahalanobis distance of each fiber segment from the fiber core. This approach has been previously used in several studies, some of the most recent in mild traumatic brain injury,(Goodrich-Hunsaker *et-al*, 2018) psychiatric(Banfi and Koschutnig, 2018; Deng *et-al*, 2018; Landerl *et-al*, 2018) and neurologic (Zhang *et-al*, 2018) studies.

**Supplemental Table-1**

| **Between-group difference in head motion during diffusion imaging acquisition time.** | | | | | | | |
| --- | --- | --- | --- | --- | --- | --- | --- |
| *MOVEMENTS PARAMETERS* | *GROUP* | *N* | *Mean* | *SD* | *t* | *df* | *Sig. (2-tailed)* |
| translation x- axis [mm] | CONT | 45 | 0.29 | 0.19 | -1.119 | * | 0.271 |
|  | OCD | 48 | 0.40 | 0.55 |  |  |  |
| translation y-axis [mm] | CONT | 45 | 0.76 | 0.36 | -1.378 | 81 | 0.177 |
|  | OCD | 48 | 1.01 | 0.96 |  |  |  |
| translation z-axis [mm] | CONT | 45 | 0.52 | 0.34 | -1.340 | 81 | 0.190 |
|  | OCD | 48 | 0.87 | 1.42 |  |  |  |
| rotation x- axis [rad] | CONT | 45 | 0.01 | 0.01 | -1.920 | 81 | 0.059 |
|  | OCD | 48 | 0.01 | 0.01 |  |  |  |
| rotation y- axis [rad] | CONT | 45 | 0.01 | 0.01 | -1.855 | * | 0.068 |
|  | OCD | 48 | 0.01 | 0.01 |  |  |  |
| rotation z- axis [rad] | CONT | 45 | 0.01 | 0.00 | -1.826 | * | 0.073 |
|  | OCD | 48 | 0.01 | 0.01 |  |  |  |
|  |  |  |  |  |  |  |  |
| ** Equal variance not assumed.* | | | | | | | |

**Supplemental Table-2**

| **Repeated measure ANCOVAs, one for each symptom dimension, MDD comorbidity or SSRI as** **the between-subjects effect, and** **FA in two segment clusters (Laterality: 2 levels including the two segment-clusters in the left and right cingulum bundle) as the within-subjects effect.** | | |
| --- | --- | --- |
| *Independent variables* | *F_[1,42]_* | *Sig.* |
|  |  |  |
| HRSD17 | 0.2 | 0.661 |
| Age [YY] | 0.1 | 0.723 |
| Sex [M/F] | 0.9 | 0.344 |
| Scanner [Trio/Prisma] | 5.7 | 0.021 |
| Laterality | 5.7 | 0.022 |
|  |  |  |
| HAMA | 0.0 | 0.861 |
| Age [YY] | 0.2 | 0.700 |
| Sex [M/F] | 0.9 | 0.355 |
| Scanner [Trio/Prisma] | 5.4 | 0.024 |
| Laterality | 4.3 | 0.045 |
|  |  |  |
| Y-BOCS | 0.3 | 0.574 |
| Age [YY] | 0.1 | 0.726 |
| Sex [M/F] | 0.7 | 0.418 |
| Scanner [Trio/Prisma] | 5.4 | 0.026 |
| Laterality | 4.6 | 0.037 |
|  |  |  |
| OCTCDQ harm avoidance | 1.9 | 0.174 |
| Age [YY] | 0.1 | 0.732 |
| Sex [M/F] | 0.6 | 0.445 |
| Scanner [Trio/Prisma] | 6.8 | 0.012 |
| Laterality | 2.6 | 0.112 |
|  |  |  |
| OCTCDQ incompleteness | 0.2 | 0.638 |
| Age [YY] | 0.1 | 0.818 |
| Sex [M/F] | 0.6 | 0.431 |
| Scanner [Trio/Prisma] | 5.6 | 0.023 |
| Laterality | 4.7 | 0.036 |
|  |  |  |
| POPS | 0.2 | 0.695 |
| Age [YY] | 0.0 | 0.968 |
| Sex [M/F] | 2.1 | 0.156 |
| Scanner [Trio/Prisma] | 5.6 | 0.023 |
| Laterality | 0.0 | 0.958 |
|  |  |  |
| MDD comorbidity [YES/NO] | 0.4 | 0.542 |
| Age [YY] | 0.6 | 0.813 |
| Sex [M/F] | 1.1 | 0.309 |
| Scanner [Trio/Prisma] | 5.6 | 0.022 |
| Laterality | 4.9 | 0.032 |
|  |  |  |
| SSRI [ON/OFF] | 0.2 | 0.663 |
| Age [YY] | 0.1 | 0.723 |
| Sex [M/F] | 0.9 | 0.344 |
| Scanner [Trio/Prisma] | 5.5 | 0.024 |
| Laterality | 4.9 | 0.032 |

| *Scanner, age and sex were covariates* |
| --- |
| *Significance level was set at 0.05/8=0.006 to account for the multiple comparisons.* |

**Supplemental Table-3A.** Corticospinal Tract – control tract

| Corticospinal Tract |  |  |
| --- | --- | --- |
|  |  |  |
| *Tests of Between-Subjects Effects* | *F^[1,87]^* | *Sig.* |
| GROUP | 2.0 | 0.160 |
| Age | 6.0 | 0.017 |
| Gender | 0.2 | 0.660 |
| SCANNER | 3.3 | 0.072 |
|  |  |  |
| *Tests of Within-Subjects Effects* | *F^[1, 87]^* | Sig. |
| LATERALITY | 0.2 | 0.677 |
| LATERALITY * GROUP | 0.1 | 0.724 |
| *Tests of Within-Subjects Effects* | *F^[4,376]^* | *Sig.* |
| LATERALITY * GROUP * SEGMENT | 0.535 | 0.722 |

**Supplemental Table-3B.** Other major white matter tracts

| Forceps Major of the Corpus Callosum | |  |
| --- | --- | --- |
|  |  |  |
| *Tests of Between-Subjects Effects* | *F^[1,88]^* | *Sig.* |
| GROUP | 2.403 | 0.130 |
| Gender | 0.988 | 0.327 |
| Age | 1.456 | 0.235 |
| SCANNER | 0.816 | 0.372 |
|  |  |  |
|  |  |  |
| *Tests of Within-Subjects Effects* | F^[1, 88]^ | Sig. |
| LATERALITY | 0.019 | 0.892 |
| LATERALITY * GROUP | 1.808 | 0.187 |
| *Tests of Within-Subjects Effects* | *F^[98,7938]^* | *Sig.* |
| LATERALITY * GROUP * SEGMENT | 0.765 | 0.883 |
|  |  |  |
|  |  |  |
|  |  |  |
| Forceps Minor of the Corpus Callosum | |  |
|  |  |  |
| *Tests of Between-Subjects Effects* | *F^[1,88]^* | *Sig.* |
| GROUP | 0.295 | 0.589 |
| Gender | 2.232 | 0.141 |
| Age | 0.405 | 0.527 |
| SCANNER | 2.522 | 0.118 |
|  |  |  |
| *Tests of Within-Subjects Effects* | F^[1, 88]^ | Sig. |
| LATERALITY | 0.001 | 0.970 |
| LATERALITY * GROUP | 0.695 | 0.407 |
| *Tests of Within-Subjects Effects* | *F^[49,3969]^* | *Sig.* |
| LATERALITY * GROUP * SEGMENT | 0.511 | 0.998 |
|  |  |  |
|  |  |  |
| Anterior Thalamic Radiation |  |  |
|  |  |  |
| *Tests of Between-Subjects Effects* | *F^[1,88]^* | *Sig.* |
| GROUP | 2.758 | 0.101 |
| Gender | 10.543 | 0.002 |
| Age | 0.797 | 0.375 |
| SCANNER | 4.230 | 0.044 |
|  |  |  |
| *Tests of Within-Subjects Effects* | F^[1, 88]^ | Sig. |
| LATERALITY | 0.935 | 0.337 |
| LATERALITY * GROUP | 2.096 | 0.152 |
| *Tests of Within-Subjects Effects* | *F^[98,7938]^* | *Sig.* |
| LATERALITY * GROUP * SEGMENT | 0.628 | 0.998 |
|  |  |  |
|  |  |  |
|  |  |  |
|  |  |  |
| Inferior Fronto-occipital Fasciculus |  |  |
|  |  |  |
| *Tests of Between-Subjects Effects* | *F^[1,88]^* | *Sig.* |
| GROUP | 0.286 | 0.595 |
| Gender | 4.221 | 0.045 |
| Age | 0.028 | 0.867 |
| SCANNER | 12.916 | 0.001 |
|  |  |  |
| *Tests of Within-Subjects Effects* | F^[1,^ ^88]^ | Sig. |
| LATERALITY | 0.353 | 0.555 |
| LATERALITY * GROUP | 0.530 | 0.470 |
| *Tests of Within-Subjects Effects* | *F^[98,7938]^* | *Sig.* |
| LATERALITY * GROUP * SEGMENT | 0.738 | 0.975 |
|  |  |  |
|  |  |  |
|  |  |  |
|  |  |  |
| Inferior Longitudinal Fasciculus |  |  |
|  |  |  |
| *Tests of Between-Subjects Effects* | *F^[1,88]^* | *Sig.* |
| GROUP | 0.064 | 0.801 |
| Gender | 1.285 | 0.262 |
| Age | 0.050 | 0.825 |
| SCANNER | 1.097 | 0.300 |
|  |  |  |
| *Tests of Within-Subjects Effects* | F^[1, 81]^ | Sig. |
| LATERALITY | 0.269 | 0.606 |
| LATERALITY * GROUP | 5.072 | 0.028 |
| *Tests of Within-Subjects Effects* | *F^[98,7938]^* | *Sig.* |
| LATERALITY * GROUP * SEGMENT ** | 1.775 | <0.001 |
|  |  |  |
| *** Adults with OCD showed lower FA that healthy adults in a middle portion (a cluster of 9 segments) of the left inferior longitudinal fasciculus. Mean FA in this cluster did not correlate with symptom severity, as measured by the HRSD, HAMA, Y-BOCS, OCIR, POPS, OCTCDQ harm avoidance and OCTCDQ incompleteness, or temperamental features.* | | |
|  |  |  |
|  |  |  |
| Superior Longitudinal Fasciculus - Parietal | |  |
|  |  |  |
| *Tests of Between-Subjects Effects* | *F^[1,88]^* | *Sig.* |
| GROUP | 0.039 | 0.844 |
| Gender | 0.022 | 0.881 |
| Age | 0.821 | 0.368 |
| SCANNER | 6.025 | 0.016 |
|  |  |  |
| *Tests of Within-Subjects Effects* | F^[1, 88]^ | Sig. |
| LATERALITY | 1.356 | 0.248 |
| LATERALITY * GROUP | 0.043 | 0.835 |
| *Tests of Within-Subjects Effects* | *F^[98,7938]^* | *Sig.* |
| LATERALITY * GROUP * SEGMENT | 0.863 | 0.830 |
|  |  |  |
|  |  |  |
|  |  |  |
| Superior Longitudinal Fasciculus - Temporal | |  |
|  |  |  |
| *Tests of Between-Subjects Effects* | *F^[1,88]^* | *Sig.* |
| GROUP | 0.404 | 0.527 |
| Gender | 0.237 | 0.628 |
| Age | 0.787 | 0.378 |
| SCANNER | 17.312 | <0.001 |
|  |  |  |
| *Tests of Within-Subjects Effects* | F^[1, 88]^ | Sig. |
| LATERALITY | 2.699 | 0.105 |
| LATERALITY * GROUP | 0.273 | 0.603 |
| *Tests of Within-Subjects Effects* | *F^[98,7938]^* | *Sig.* |
| LATERALITY * GROUP * SEGMENT | 1.289 | 0.030 |
|  |  |  |
|  |  |  |
| Uncinate Fasciculus |  |  |
|  |  |  |
| *Tests of Between-Subjects Effects* | *F[^1,88]^* | *Sig.* |
| GROUP | 0.002 | 0.965 |
| Gender | 0.883 | 0.350 |
| Age | 0.006 | 0.938 |
| SCANNER | 14.287 | <0.001 |
|  |  |  |
| *Tests of Within-Subjects Effects* | F^[1, 81]^ | Sig. |
| LATERALITY | 1.883 | 0.174 |
| LATERALITY * GROUP | 0.348 | 0.557 |
| *Tests of Within-Subjects Effects* | *F^[98,7938]^* | *Sig.* |
| LATERALITY * GROUP * SEGMENT | 0.921 | 0.696 |

**Supplemental Table-4.**

| **Two Factorial Repeated Measure ANCOVA in the cingulum bundle in 40 adults with OCD and 41 healthy adults:** Sensitivity Analysis [PRISMA SCANNER]. | | |
| --- | --- | --- |
| *Tests of Between-Subjects Effects* | *F_[1,77]_* | *Sig.* |
| **GROUP** | **4.5** | **0.037** |
| **Age [YY]** | **7.4** | **0.008** |
| Sex [M/F] | 1.3 | 0.256 |
|  |  |  |
|  |  |  |
| *Tests of Within-Subjects Effects* | *F_[1, 77]_* | *Sig.* |
| **LATERALITY** | 0.2 | 0.689 |
| LATERALITY * GROUP | 0.2 | 0.620 |
|  |  |  |
| *Tests of Within-Subjects Effects* | *F_[98, 7546]_* | *Sig.* |
| **SEGMENT** | **12.0** | **<0.001** |
| SEGMENT * GROUP | 1.2 | 0.312 |
|  |  |  |
|  |  |  |
| *Tests of Within-Subjects Effects* | *F_[98,7546]_* | *Sig.* |
| **LATERALITY * GROUP * SEGMENT** | 1.2 | 0.277 |
|  |  |  |

*Age and gender were covariates in all analyses*

*Two factorial analysis included: 1. LATERALIRY (2 levels: left and right) and SEGMENT (100 levels)*

**References.**

Banfi C, Koschutnig K (2018). White matter alterations and tract lateralization in children with dyslexia and isolated spelling deficits.

Caruyer E, Lenglet C, Sapiro G, Deriche R (2013). Design of multishell sampling schemes with uniform coverage in diffusion MRI. *Magnetic resonance in medicine : official journal of the Society of Magnetic Resonance in Medicine / Society of Magnetic Resonance in Medicine* **69**(6): 1534-1540.

Deng F, Wang Y, Huang H, Niu M, Zhong S, Zhao L*, et al* (2018). Abnormal segments of right uncinate fasciculus and left anterior thalamic radiation in major and bipolar depression. *Prog Neuropsychopharmacol Biol Psychiatry* **81**: 340-349.

Ecker W, Gonner S (2008). Incompleteness and harm avoidance in OCD symptom dimensions. *Behav Res Ther* **46**(8): 895-904.

Ecker W, Gonner S, Wilm K (2011). [The measurement of motivational dimensions of OCD: incompleteness and harm avoidance]. *Psychother Psychosom Med Psychol* **61**(2): 62-69.

Ettelt S, Grabe HJ, Ruhrmann S, Buhtz F, Hochrein A, Kraft S*, et al* (2008). Harm avoidance in subjects with obsessive-compulsive disorder and their families. *J Affect Disord* **107**(1-3): 265-269.

Goodman WK, Price LH, Rasmussen SA, et al. (1989). The yale-brown obsessive compulsive scale: Ii. validity. *Archives of general psychiatry* **46**(11): 1012-1016.

Goodrich-Hunsaker NJ, Abildskov TJ, Black G, Bigler ED, Cohen DM, Mihalov LK*, et al* (2018). Age- and sex-related effects in children with mild traumatic brain injury on diffusion magnetic resonance imaging properties: A comparison of voxelwise and tractography methods. *Journal of neuroscience research* **96**(4): 626-641.

Hamilton M (1959). The assessment of anxiety states by rating. *Br J Med Psychol* **32**(1): 50-55.

Hamilton M (1960). A rating scale for depression. *Journal of Neurology, Neurosurgery and Psychiatry* **23**: 56-62.

Landerl K, Lin Q, Bu X, Wang M, Liang Y, Chen H*, et al* (2018). Aberrant white matter properties of the callosal tracts implicated in girls with attention-deficit/hyperactivity disorder. *Hum Brain Mapp*.

Lyoo IK, Lee DW, Kim YS, Kong SW, Kwon JS (2001). Patterns of temperament and character in subjects with obsessive-compulsive disorder. *J Clin Psychiatry* **62**(8): 637-641.

Pietrefesa AS, Coles ME (2008). Moving beyond an exclusive focus on harm avoidance in obsessive compulsive disorder: considering the role of incompleteness. *Behav Ther* **39**(3): 224-231.

Wakana S, Jiang HY, Nagae-Poetscher LM, van Zijl PCM, Mori S (2004). Fiber tract-based atlas of human white matter anatomy. *Radiology* **230**(1): 77-87.

Zhang J, Wei X, Xie S, Zhou Z, Shang D, Ji R*, et al* (2018). Multifunctional Roles of the Ventral Stream in Language Models: Advanced Segmental Quantification in Post-Stroke Aphasic Patients. *Front Neurol* **9**: 89.
